# Defective Speech

**Published:** 1887-10

**Authors:** 


### DEFECTIVE SPEECH.

A child with a cleft palate uses his tongue and the muscles of the palate in an unnatural way. Hence, after the closure of the cleft by an operation, an impediment remains, because the patient is still controlled by his previous habits. He will need, therefore, systematic training for months or years, to acquire right habits in the case. One prime requisite is, that he fix his entire attention on his teacher, and slowly imitate him, as the latter strongly pronounces the difficult words, making every movement of his lips and tongue as plain as possible. The palate may be so highly arched that the child's speech is not much better than that of the former case. But the soft part of the palate can be lowered by an operation, and the speech greatly improved by training. There may be extreme backwardness in speaking, though there is no organic defect. Here, a practiced eye will see that there is mental feebleness. Speech training will be a part of the necessary training of the intellectual powers.

In other cases, the mental powers generally may be good, and the child be indeed specially bright, but, for some cerebral reason, he lisps badly, or finds it difficult to pronounce the letters *s* or *l*, or he speaks in a babyish way. Such patients will generally overcome the difficulty in time. Sometimes the voice is "stuffy," especially in endeavoring to utter *m*, *n*, or *ng*. The word *morning* sounds like *bordig*. This is due to growths in the nose or back mouth. On the removal of these, by a surgical operation, the voice recovers its normal character. Enlarged tonsils sometimes cause a somewhat similar difficulty. Another defect is stammering. Usually the person has a nervous constitution, and has been subjected to some nervous strain, generally at school. In such cases, all disturbing causes should be removed, and the nervous system be invigorated as far as possible. But the stammerer must be taught to speak with deliberate enunciation, imitating the teacher in the utterance of all difficult words, and especially filling the lungs well at every stop—*London Lancet.*

